# Monoscopic photogrammetry to obtain 3D models by a mobile device: a method for making facial prostheses

**DOI:** 10.1186/s40463-016-0145-3

**Published:** 2016-05-25

**Authors:** Rodrigo Salazar-Gamarra, Rosemary Seelaus, Jorge Vicente Lopes da Silva, Airton Moreira da Silva, Luciano Lauria Dib

**Affiliations:** UNIP Postgraduate Dental School, Universidade Paulista, Rua Afonso Braz, 525 - Cj. 81 Vila Nova Conceição, São Paulo, CEP 04511-011 SP Brazil; The Craniofacial Center, University of Illinois at Chicago, 811 S Paulina St, Chicago, IL 60612 USA; Division of the Centro Tecnológico da Informação Renato Archer, Rodovia Dom Pedro I, Km 143, 6 - Amarais, Campinas, SP 13069-901 Brazil; Centro Tecnológico da Informação Renato Archer Campinas, Rodovia Dom Pedro I, Km 143, 6 - Amarais, Campinas, SP 13069-901 Brazil; Oncology Center, Hospital Alemão Oswaldo Cruz, Rua Afonso Braz, 525 - Cj. 81 Vila Nova Conceição, São Paulo, CEP 04511-011 SP Brazil

**Keywords:** 123D Catch, 3D photography, Maxillofacial rehabilitation, Facial prosthetics, Photogrammetry, Oral rehabilitation

## Abstract

**Purpose:**

The aim of this study is to present the development of a new technique to obtain 3D models using photogrammetry by a mobile device and free software, as a method for making digital facial impressions of patients with maxillofacial defects for the final purpose of 3D printing of facial prostheses.

**Methods:**

With the use of a mobile device, free software and a photo capture protocol, 2D captures of the anatomy of a patient with a facial defect were transformed into a 3D model. The resultant digital models were evaluated for visual and technical integrity. The technical process and resultant models were described and analyzed for technical and clinical usability.

**Results:**

Generating 3D models to make digital face impressions was possible by the use of photogrammetry with photos taken by a mobile device. The facial anatomy of the patient was reproduced by a *.3dp and a *.stl file with no major irregularities. 3D printing was possible.

**Conclusions:**

An alternative method for capturing facial anatomy is possible using a mobile device for the purpose of obtaining and designing 3D models for facial rehabilitation. Further studies must be realized to compare 3D modeling among different techniques and systems.

**Clinical implication:**

Free software and low cost equipment could be a feasible solution to obtain 3D models for making digital face impressions for maxillofacial prostheses, improving access for clinical centers that do not have high cost technology considered as a prior acquisition.

## Background

Facial mutilation and defects could derive from cancer, tumors, trauma, infections, congenital or acquired deformation and affect quality of life due to the impact on essential functions such as communication, breathing, feeding and aesthetics [1–5]. Rehabilitation of these patients is possible with adhesive-retained facial prosthetics, implant supported facial prosthetics and plastic surgery [2, 6–12]. Although some aesthetic results can be achieved by plastic surgery [13, 14], frequently this requires multiple attempts which are time consuming and costly [15]. In most cases worldwide, defects of external facial anatomy are primarily treated by prostheses [16, 17]. Still, for the realization of a prosthesis, a highly trained and skilled specialist is required to sculpt a form mimicking the lost anatomy, and to handle the time-consuming technical fabrication process.

To make a facial prosthesis, an impression is required to record the anatomic area of the defect. Some impression materials have demonstrated high and accurate precision registering details of defects and the surrounding anatomy [18–21], but present other difficulties and limitations [22, 23]. Some challenges are related to the technical sensitivity of the material, working time and setting time. Training and experience is needed to handle the materials, especially when working near the airway, and frequently require the assistance of a second professional to help in the procedure. In cases of large facial defects, there is a need to cover all the face which can be claustrophobic for the patient. Also the weight of the materials and the use of cannulas, to allow free airway during the procedure with the mouth opening, can deform the residual facial tissues, causing distortion in the impression [22]. The economic cost of large usage of impression materials is also a topic of concern. A limitation of conventional facial impressions is that they cannot predict information about results of the final rehabilitation because they only register detail of the defect and surrounding tissues.

To address these difficulties of conventional facial impressions, some authors reported [24, 25] clinical cases using Computerized Tomography (CT-Scan) [26, 27], Magnetic Resonance Imaging (MRI) [27, 28], Laser impressions [27, 29–32] and 3D photography [33, 34] to record extra-oral digital impressions. Digital impressions are also used to print working models [34], design prostheses digitally by mirroring from a healthy side [36], digitally capturing structures from a healthy donor patient [37]^,^ for designing templates of the final prostheses and prototyping it, or to design a prototype model of the flask where the silicone is directly packaged [31, 38]. These reports represent a viable way to rehabilitate patients in less time, with more effectiveness, improved accuracy and less effort by the patient and the professional. However the use of such technologies can produce even higher costs in software, hardware or other equipment. Different authors have sought alternatives to transform these impressions with low cost solutions [38], but there is still no consensus nor a concept widely accepted.

Among all the possible methods for 3D surface imaging and data acquisition, 3D photogrammetry is attractive for its capacity to obtain 3D models from 2D pictures, the capture and process speed, absence of radiation for patient, good results and non-complex training [39–41]. 3D photography is performed by a method called photogrammetry, that emerged from radiolocation, multilateration and radiometry and it has been used since the mid-19th century in industries of space, aeronautics, geology, meteorology, geography, tourism, and entertainment. More recently, applications in general medicine have been reported. Photogrammetry allows “Structure from Motion” (SFM) where the software examines common features in each image and is able to construct a 3D form from overlapping features, by a complex algorithm that minimizes the sum of errors over the coordinates and relative displacements of the reference points. This minimization is known as “bundle adjustment” and is often performed using the Levenberg-Marquardt algorithm. Photogrammetry can be used in a stereophotogrammetry technique, where all captures are made simultaneously by different cameras at different heights and angles relative to the object/subject; or, by a monoscopic technique, where only one camera is used to do sequential captures at different heights and angles from to the object/subject [39–41]. This industry has developed different products and systems for simplifying the clinical application obtaining increasingly better results. On the other hand, this technology demands high costs for hardware, software and infrastructure and may not be possible for many centers worldwide.

Alternatives for expensive photogrammetry are free software that can be used by computers, tablets and other mobile devices to generate 3D models from 2D pictures by similar methods (Autodesk 123D Catch®, California, US) [42, 43]. Initially, the target of these software was entertainment and other non-medical use. Recently Mahmoud [42] used this free software for medical educational reasons and Koban [43] for making an evaluation and analysis for plastic surgery planning on a plastic mannequin. To the authors best knowledge, monoscopic photogrammetry has not been published for facilitating the process of fabrication of facial prostheses in humans, by adapting this low cost technology into a clinical solution. The possibility to decrease the cost of fabrication of facial prostheses with the use of mobile devices and free software would warrant investigation for the benefit of most parts of the world.

The incorporation of technology into the fabrication process of facial prostheses has the potential to transform the rehabilitation, from a time-consuming artistically driven process to a reconstructive biotechnology procedure [24]. One of the methods for surface data acquisition and 3D modeling is 3D photography (photogrammetry) that has been used in medical sciences since 1951[44–46]. In recent years, techniques and methods have been improving to the benefit of the surgical and prosthetic team [47, 48]. Technical validation and evaluation of sophisticated photogrammetry systems have reported beneficial applications in facial prosthetic treatment [49–57]. 3D photography has been a practical solution in clinical practice compared with other 3D model obtaining methods (MRI, CT-Scan & Laser) [26–37, 58–64]. Still this technology requires substantial investment in infrastructure, hardware and software for clinical practice [65, 66]. For this reason, some authors have pursued low cost processes for fabrication of facial prosthetics [38], with the use of free software and the monoscopic photogrammetry technique with mobile devices [42, 43].

## Methods

### Patient selection

One subject, who attended the Maxillofacial Prosthetic Clinic of the *Universidade Paulista* in *São Paulo* for prosthetic rehabilitation, was selected after being advised about ethical aspects of the research and freely accepted to participate. Informed consent was obtained from the patient.

### Data acquisition

#### Subject and operator positioning

The subject was positioned in a 45 cm-high chair in an upright seated position, with 1 meter of floor space between the chair and the position of the operator with 0° – 180° of clearance laterally, where 90° was the primary area of interest to capture. Floor clearance allowed sufficient room for the operator to move around the subject during the capture process. An adjustable-height (30 cm to 50 cm) chair with wheels for mobility was used by the operator. Earrings, hats, glasses or other accessories that could interfere with the area of capture were removed from the subject prior to photo capture. The subject was instructed to remain still in order to eliminate balance movement and maintain the head in an orthostatic position with the Frankfurt plane parallel to the floor. If balance of the head was detected after giving the instruction of not moving, a head support was used between the head and a wall. The subject was also instructed to: maintain a neutral facial expression, with jaw and lips closed without force (maximal intercuspal occlusion); to wear his intraoral removable prostheses for giving support to the facial tissues; and, to blink between photographs repeating the same eye position. Visual color contrast between the background and the colors of the skin and hair of the subject was established. A clinical measurement of the inter-alar nasal distance was registered for further scale verification.

#### Lighting

Sufficient lighting in the room was ensured such that the ambient light enabled taking clear images without flash and without underexposing or overexposing images. Lights of the room, blinds and curtains of the windows were opened and orientation of the ambient light was considered to avoid getting shadows on the area of interest through the process of capture. Irregular lighting was avoided, like strong back-lighting and direct, intense light to the subject. Objects with strong reflective or shiny surfaces were eliminated from the camera’s field of view during the photo capture process.

#### Mobile device and application

An internet Wi-Fi 5Ghz network connection was used. A free photogrammetry application (Autodesk 123D Catch®, California, US) was downloaded by a mobile device (Samsung Galaxy Note 4® - Seoul, South Korea) through the Android® Google Playstore® (California, US). A 123D Catch® and a free account was created. All automatic features of the mobile device were enabled as needed by the application for the data acquisition process. Features of the mobile device are outlined in (Table 1). 123D Catch® PC version was also downloaded in a Windows PC (Dell Inspiron 1525 Dual Core).

**Table 1:** Mobile Device technical features

Samsung galaxy note 4 - Brasilian standard version, software actualized at 10/03/2015
Hardware & Software
1. Model: SM-N910C
2. Android version 4.4.4
3. Kernel version 3.10.9-3317155 (Fundamental software of the operating system)
4. KNOX version 2.2 (Informatic security)
5. 2.7GHz Quad Core Process, 1.9GHz Octa Core (1.9GHz Quad + 1.3GHz Quad Core) Process
6. MEMORY 3GB RAM + 32GB Internal memory
7. NETWORK 2.5G (GSM/GPRS/EDGE) : 850/900/1800/1900 MHz. 3G (HSPA+ 42Mbps): 850/900/1900/2100 MHz, 4G (LTE Cat.4 150/50Mbps) or 4G (LTE Cat.6 300/50Mbps)
8. CONNECTIVITY Wi-Fi 802.11 a/b/g/n/ac (2X2 MIMO)
9. Camera F1.9 lens camera and 16MP Smart OIS, 31 mm focal length
10. Accelerometer sensor (identify the position and movement of the cellphone and registers data in axis X, Y & Z)
11. Gyroscope sensor (Identify the status of rotation of the telephone in axis X and Y)

#### Photo capture

**Fig. 1:**
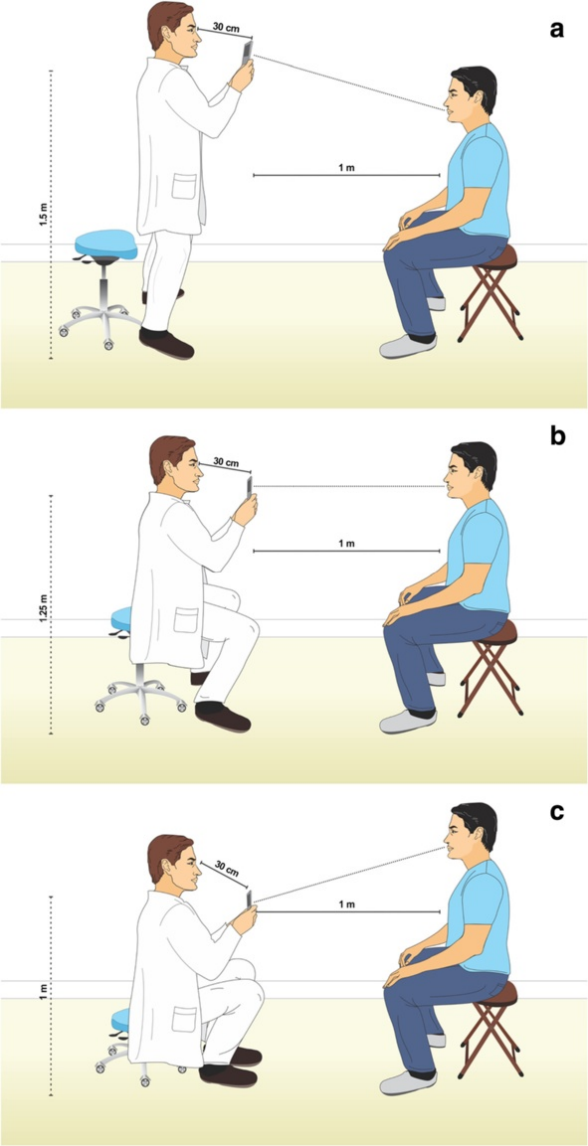
**a**. Simulation of the Height 1, where the operator is at a stand up height and maintain the mobile device 30 cm from his head, 1.5 m from the floor and 1 meter from the patient. **b**. Simulation of the Height 2, where the operator sits on the higher height of the chair with wheels and maintain the mobile device 30 cm from his head, 1.25 m from the floor and 1 meter from the patient. **c**. Simulation of the Height 3, where the operator sits on the lower height of the chair with wheels and maintain the mobile device 30 cm from his head, 1 m from the floor and 1 meter from the patient

**Fig. 2:**
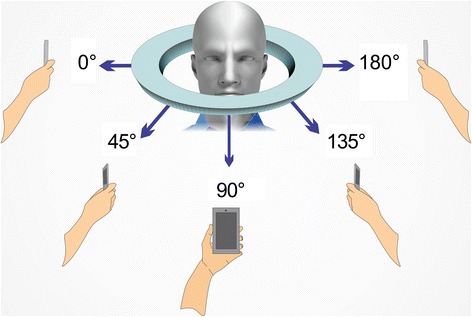
Simulation of angles of photo captures per each height

**Fig. 3:**
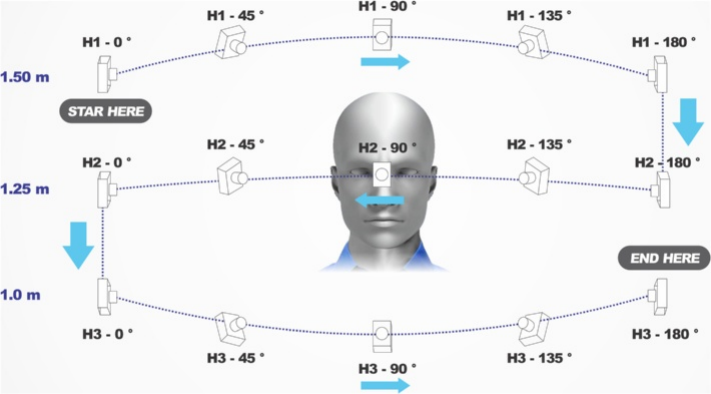
Simulation of the complete sequence of photo capture protocol around the area of interest for capture

**Fig. 4:**
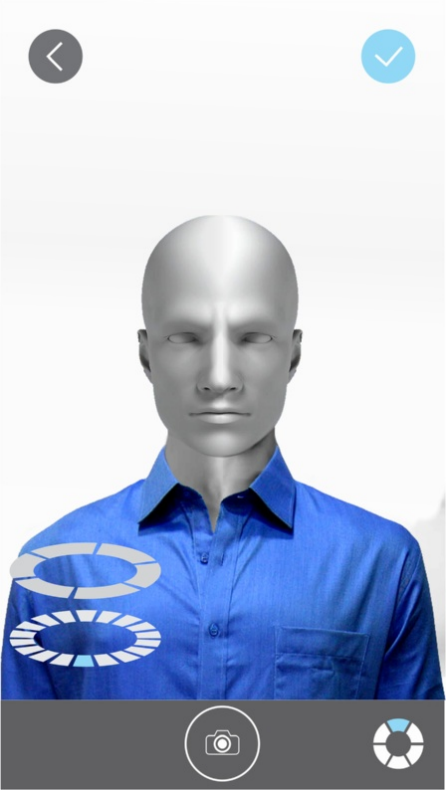
Mobile device screen simulation with the patient in a H2-90° position and 1 meter distance from subject and camera. Image also shoes the “Check” button up in the right side, positioning gadget down in the left side and the photo capture shooting button down in the middle

The photogrammetry application was opened from the mobile device and new capture was selected by pressing the “+” button in the upper right corner. A planned sequence of 15 conventional 2D photos were taken, always with the area of interest for capturing as the center of the picture and with the operator maintaining a 30 cm distance between his eyes and the mobile device, raising it up to his same eye-height position. Photo captures were taken at three different heights. The first height (H1) was the standup-height of the operator (1.75 m) with the mobile device at 1.50 m of height from the floor. (Figure 1a) The second height (H2) was with the operator seated on the moveable chair at its maximum adjustable height (50 cm) and maintaining the mobile device at 1.25 m from the floor. (Figure 1b) The third height (H3) was with the operator seated on the same chair at its lowest adjustable height (30 cm) with the mobile device at 1 m of height above the floor. (Figure 1c) Each height repeated the same positions for taking the photo captures and was taken at the 0°, 45°, 90°, 135° and 180°, considering 0° as subject’s right side, 90° as the midline of the face and 180° as the subject’s left side (Fig. 2). All photo captures were perpendicular to the primary area of interest. The operator took the first picture starting from H1-0° at a one meter distance from the subject. The complete sequence was H1-0°, H1-45°, H1-90°, H1-135° H1-180°, H2-180°, H2-135°, H2-90°, H2-45°, H2-0°, H3-0°, H3-45°, H3-90°, H3-135° and H3-180°, completing the 15 photo captures (Fig. 3). For photo capture, the “autofocus” was used at the center of the area of interest, avoiding blurry photographs. The “position-in-space-recognition gadget” of the application was used to guide the position of photo captures and to register total numbers of photos recorded in the process (Fig. 4). Following the photo capture, the operator reviewed the integrity of each picture, verifying that there were no illumination irregularities, blurry images, incomplete parts of the face of the subject or any other evident errors in the picture that would compromise data processing. After ensuring the good quality of the photo captures, the subject was released from his static position and the “check” button was pressed for uploading the pictures for processing.

#### Photo capture review and 3D processing

When all 15 photo captures were taken, the “check” button in the upper right corner of the application was pressed and captures were shown in the visor to be reviewed and approved with another pressing of the “Check” button. The application started automatically to upload and process the captures into the 123D Catch® servers. Once finished, the digital model was reviewed through the mobile device to verify its integrity.

All photo captures taken by the mobile device were downloaded from 123D Catch® website and meshed through the 123D Catch® PC version with the maximum quality of meshing. A *.3Dp and *.stl files were obtained. The *.3Dp file was opened and reviewed from 123D Catch® PC version for primary analyzing and the *.stl file was opened and edited from Autodesk Meshmixer (California, US). Editing in Meshmixer® only considered model repositioning in space (x-y-z axis transform tool) into a straight position, deleting triangles beyond the face and re-scaling model into the inter-alar nasal distance that had been clinically registered. 360° degrees observation and in all x-y-z axis angles for descriptive analysis was performed and the model of the face of the patient was printed in Duraform Polyamide C15 degraded material by a Sinterstation HiQ by Selective Laser Sinterization (SLS) (3D Systems, Rockhill SC, USA).

## Results

**Fig. 5:**
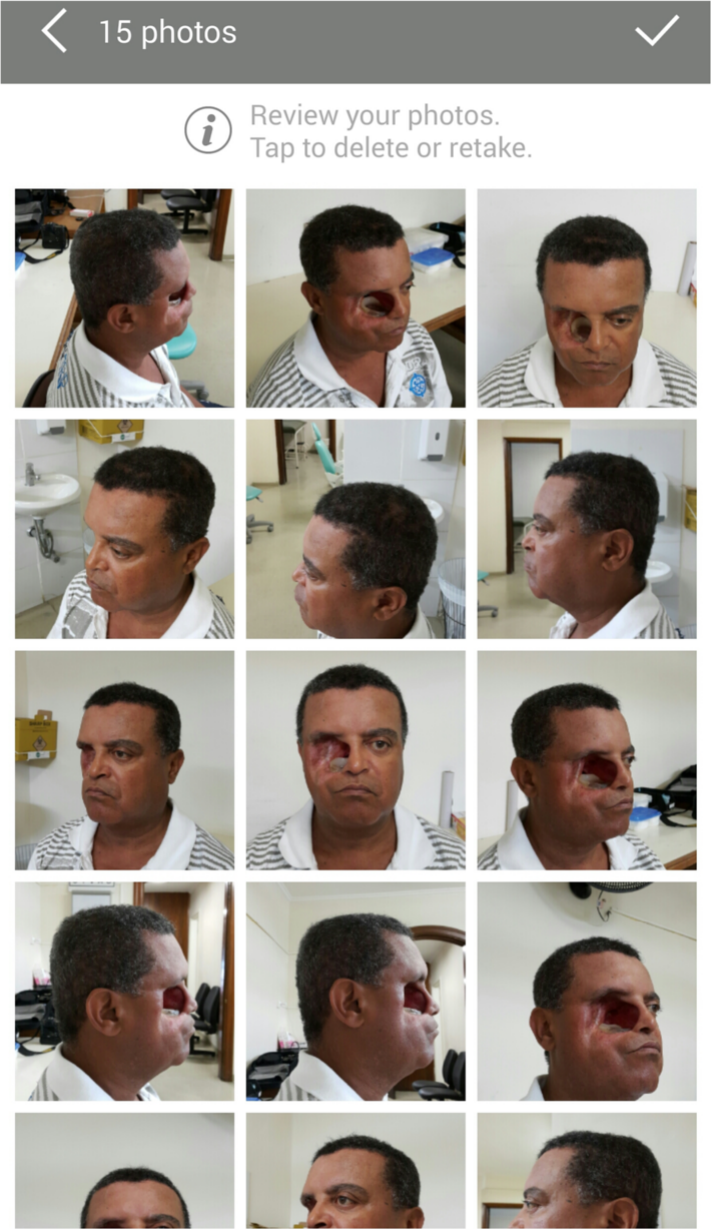
Mobile device screen with the 15 photo captures of the patient in the sequence of our protocol

**Fig. 6:**
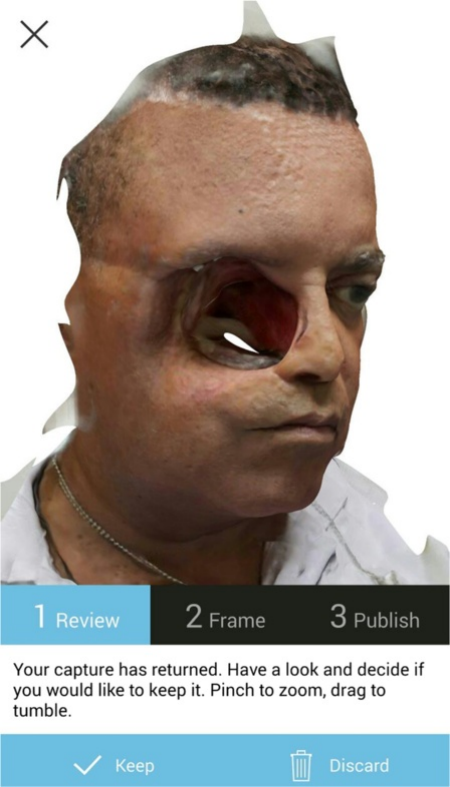
Screen of the mobile device with the 123D Catch® Mobile App model-reviewing, after the upload and meshing in the Autodesk servers

With the use of 123D Catch® mobile device application using the described photo capture protocol, fifteen two-dimensional colored photo captures were obtained in *.jpeg file format. Automatically, according to the mobile device camera features, sizes of photo captures varied from 4710 kb to 5931 kb with an average size of 5118 kb. Revision of the captured photos before processing detected that all captures were compatible with the protocol (Fig. 5). The revision of the created digital model through the mobile device before downloading found no major irregularities which could interrupt the process (Fig. 6).

**Fig. 7:**
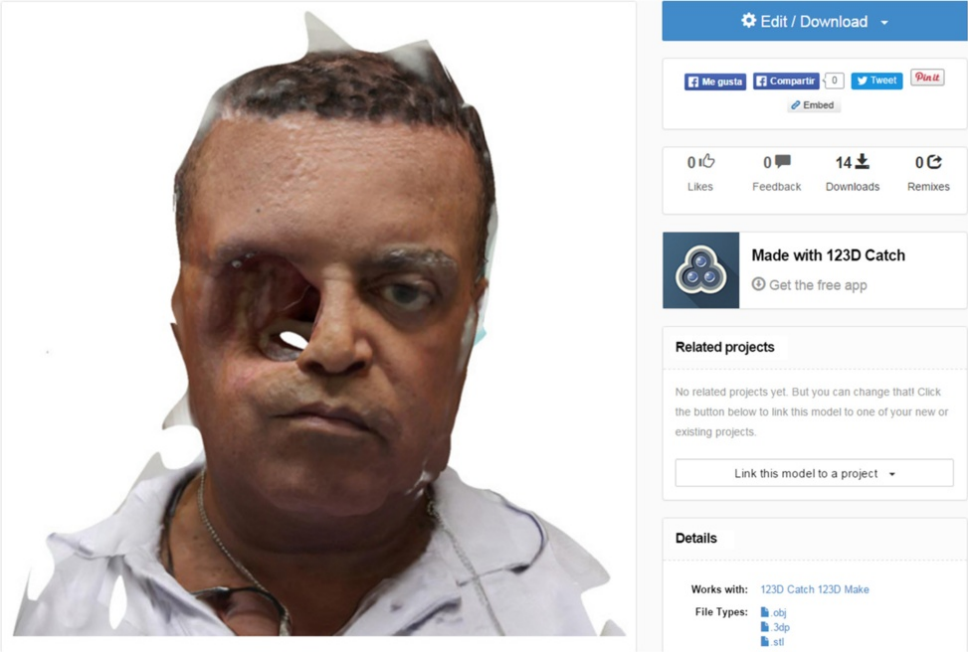
123D Catch® Web model reviewing and where files were downloaded

**Fig. 8:**
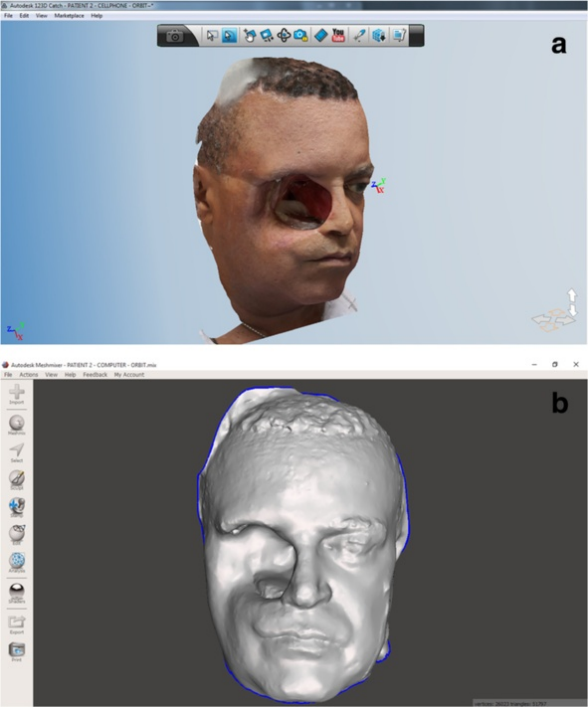
**a**. 123D Catch® PC version review of the *.3Dp model. **b**. Screen capture of Meshmixer® reviewing the *.stl model after setting up-right position, rescaling and deleting triangles beyond the face

Digital model and photo captures were downloaded from the Autodesk webpage. Photo captures were re-processed in high quality through the 123D Catch® PC version (Fig. 7). The combined use of 123D Catch® mobile device application and pc version created high quality *.3Dp and *.stl files from the 15 individual 2D photographs, with file sizes of *.3Dp and *.stl of 5 kb and 39,918 kb respectively (Fig. 8a, b).

**Fig. 9:**
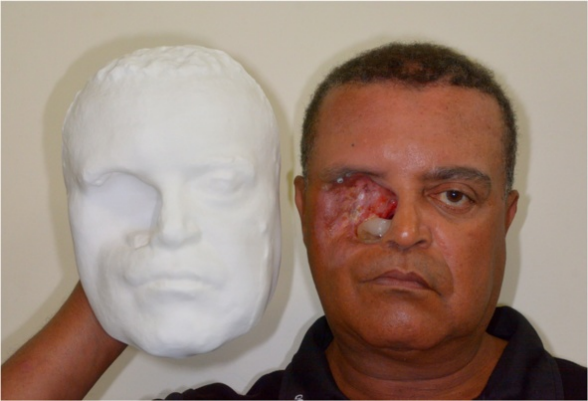
Shows the Duraform Polyamide C15 degraded material, for the impression of the model of the patient, with the patient holding it

By the use of Meshmixer®, it was possible to manually eliminate the triangles beyond the head, to reposition in space and to scale the digital model. This final manipulated digital model obtained appropriately represented the shape and proportions of the original face of the patient, leading to a printed polyamide model which also showed similarity of representation; although, some minor irregularities were detectable in the surface of eyebrows, hair and lateral sides of the patient (Figs. 8b and 9).

## Discussion

This study aimed to develop a technique to obtain 3D models by mobile device photogrammetry and the use of free software as a method for making facial impressions of patients with facial defects for the final purpose of 3D printing of facial prostheses. For this purpose a patient that voluntarily accepted to participate in the study was submitted to the proposed protocol and methods. Captures were taken by the use of 123D Catch through a mobile device by a controlled sequence, illumination and position of the operator and patient.

The rational for using a cellphone for making photo captures through the 123D Catch® application was that all modern mobile devices have an integrated accelerometer and a gyroscope sensor, which are automatically run by the application to guide the operator in a 3D position during the photo capture sequence. Also in today’s market, mobile devices are equipt with faster processors, fast network and connection qualities, high quality cameras and added features, (Table 1), at a reasonable cost to the consumer as a personal tool, and not as a clinical equipment. Monoscopic photogrammetry has been used with different kinds of cameras like SLR, prosumer, point and shoot, mobile devices and others, principally for non-medical reasons [67], but also recently, for medical purposes [42, 43].

Developers of 123D Catch® published through their web tutorials some general indications for the photo capture process, and for that reason, in the present study, a standardized sequence protocol of photo capturing was designed into a user-friendly sequence, which satisfies both the requirements of 123D Catch® and the clinical needs for maxillofacial rehabilitation. The most important considerations are sequence and orientation of capture, illumination, subject and operator positioning and clinical measurement of a stable reference of the subject. This free photogrammetry application recognizes patterns between captures that have more than 50 % of overlap between each capture [67]. For this reason, it was decided to make a sequence with 45° degree intervals between captures at each height, demonstrating acceptable results in the meshing process. If the illumination pattern is different between each capture, or the subject does not keep still during captures, or if photos are taken randomly or arbitrarily, the photo capture overlapping by the algorithm may not be possible, and will show defects, affecting the viability of using the model. It is for this reason that the flash is not used, and rotating the patient on his own axis is not recommended. Flash will generate its own pattern between each capture, and if the patient is rotated on his own axis during capture, the illumination pattern over the patient and background will differ among captures and will be unreadable by the software [67]. The ideal is to complete multiple captures, as stereophotogrammetry does, while maintaining the position of the patient during the complete sequence of photo captures, one by one, with consistent conditions of ample indirect ambient light. The position of the operator is equally important to allow capture of the entire area of interest without losing detail from too great a distance, or producing shadows by being too close to the patient. One meter of distance between the subject and the camera is compatible with aforementioned technical requirements. Distance and position are important in the capture protocol, but absolute exactness is not critical since the application still recognizes patterns with consistent light reflection [67]. Currently, no information is available about a tolerance of acceptable variance in photo capture, and how this might impact the meshing process. While there are not objective protocols for evaluating the model, the clinician must subjectively evaluate the model to see if it is below a threshold of being usable. The time-consuming process of photo capture is prone to have some irregularities [43]. In this workflow, the 3D position of the reproduced anatomy is a very well startup for sculpture. All possible errors and small texture details may not have much importance because the digital model of the prostheses will serve to produce a prototype that will be duplicated in a wax for final handwork to obtain a sculpture with finishing details, texture, and adaptation into the patient. That’s why small digital discrepancies on surface will not affect the final result of the definitive prosthesis. Actual technology, neither the expensive stereophotogrammetry systems, have not the enough imaging detail to reproduce skin texture, expression lines of the patient or others, resulting in a mandatory handwork finishing sculpture. A clinical measurement is needed for registration because 123D Catch® generates a reduced model and this is not unexpected since the application was meant for entertainment and desktop 3D printing objectives. Subsequently, scaling is required and a reliable, stable distance must be used. In our subject, the inter-alar distance of the nose was used. In other patients that have both eyes, the inter-canthal or inter-pupilar distance could be to ensure stable measurement. Small ruler or fiducial markers fixed on the patient could be used for registration and scaling purposes.

Once the models were obtained (*.3Dp & *.stl), *.3Dp models showed good appearance in color and proportions of the subject through the 123D Catch® mobile device app and PC version. The *.3Dp file was useful only by this application but can be exported as other file types like *.obj or *.stl. Alternatively, multiple file types can be directly downloaded from the web, as was done in this study. Reviewing this file on the PC version provides the colored model, which can be helpful to show to the patient, and for explanation and education of the anatomy and planning. It also provides an indication of the quality of the meshing. If substantial errors were found in this step, they were more evident in the *.stl version. Through the PC version of 123D Catch, it is possible to press the “print” button and that will take you to the *.stl in Meshmixer®, or it is possible to open the *.stl file directly from Meshmixer® as was done in the present study. Once opened the model needed to be up righted, repositioned, and rescaled according to the clinical measurement previously recorded. It was then edited to eliminate all the background and body parts of the model, which are beyond the area of interest for capture.

In the present study the models generated by the mobile device were not used directly for 3D printing. Instead, the captures made by the mobile device were meshed through the PC version of 123D Catch®. They showed better results in the surface of the models virtually and were therefore selected for printing purposes. Further studies should be conducted to better evaluate the accuracy of the respective virtual models. The PC version of 123D Catch® has an option to re-mesh the model with higher quality than the originally configured application for mobile devices. The application was not originally created for medical purposes, but rather, for more simplified CAD designs; complex organic shapes of anatomical models represent a heavier burden for mobile applications, and would run more slowly on smartphones [67].

This *.stl file showed a very acceptable replica of the anatomy of the patient. Once it was re-scaled and printed it showed that it subjectively met the needs for facial prosthetic fabrication, but further studies are needed to evaluate the precision and accuracy of this process.

While not a part of the objective of this study, once an *.stl of the patient is acquired through this process, that sufficiently recreates the anatomy, a digital prostheses design is possible. This is possible through the manipulation of the healthy side of the patient by selecting, isolating, duplicating, mirroring, transforming, editing and sculpting up to have an adequate adaptation of the prostheses model using Meshmixer®. The virtually designed prosthesis model would need to be extruded to provide a volume from the surface data, to produce the final prosthesis design for printing.

Mahmoud, et al., demonstrated that three-dimensional printing of human anatomic pathology specimens is achievable by the use of 123D Catch® and recognize that advances in 3D printing technology may further improve [42]. Koban et al. founded in a comparison between Vectra® and 123D Catch® on a labeled plastic mannequin head with landmarks, that no significant (*p* > 0.05) difference was found between manual tape measurement and digital distances from 123D Catch® and Vectra®. Also they describe that sufficient results for the 3D reconstruction with 123D Catch® is possible with 16, 12 and 9 photo captures, but with higher deviations on lateral units than in central units. Also they found that 123D Catch® needed 10 minutes on average to capture and compute 3D models (5 times more than Vectra) [43]. The present study obtained similar results in the lateral views of our models, with more irregularities compared to the primary area of interest to be captured (center of the face). This phenomenon could be associated with less intersection of overlapping triangles in those areas which are not the primary area of interest to be captured. Time was not measured as a variable of our study, but we experienced that during the automatic software uploading, meshing and downloading process, the operator’s attention could be dedicated to other tasks.

While the technology process does not print the final adapted prosthesis, some small errors in the surface of the model are acceptable, because a finishing work by hand on the wax replica of the prototyped prosthesis will be done chairside which will eliminate any “stair-stepping” from printing, ensuring appropriate adaptation to the skin surface and applying naturalistic surface texture. While finishing work in the clinic and laboratory is still required, this protocol provides a very helpful advancement in the macro-sculpture of the prosthesis, which can be tested and adapted as needed directly on the patient.

Prolonged capture time with multiple pictures is prone to errors [43] and it is for this reason that standardizing a photo capture protocol for data capture and processing is essential. A standardized photo capture protocol will simplify the process of capture-to-print-prototyping (CPP). 123D Catch® computed models suggest good accuracy of the 3D reconstruction for a standard mannequin model [43] and so is demonstrated in this study for a maxillofacial prosthetic patient.

## Conclusion

It was possible to generate 3D models as digital face impressions with the use of monoscopic photogrammetry and photos taken by a mobile device. Free software and low-cost equipment are a feasible alternative for capturing patient facial anatomy for the purpose of generating physical working models, designing templates for facial prostheses, improving communication with patients before and during treatment and improving access to digital clinical solutions for clinical centers that do not have high cost technology allowances in their budget. Further studies are needed to evaluate quality variables of these models. Clinical data capture protocols like the one described in this report must be validated clinically to optimize the process of data acquisition.
